# Items

**Published:** 1900-05

**Authors:** 


### ITEMS.

PARIS AND THE MIDNIGHT SUN.—A trip to the thirteenth International Medical Congress and some scenery is planning under the auspices of a committee of prominent medical men representing all sections of our country.

An itinery has been prepared after careful consultation with members proposing to be of the party who are old travelers and have been over the ground. The particular advantages of route chosen are the trip across the Atlantic on the high class, twin-screw, vessel "Campania" of the express service, the view of Scotland at the best season of the year, the sight of the Norwegian fjords, and the interesting carriage rides between Christiania and Bergen on the return from the North Cape. At that spot, the most northern extremity of Europe, the marvelous spectacle is to be seen at that season of the year of the sun shining brightly at the dead of night and making a complete circuit of the visible heavens in the twenty-four hours. Returning from the North Cape the trip is pursued to Bergen, thence to Hull, London, The Hague, Rotterdam, Brussels and Paris in time for the opening of the session of the International Medical Congress, August 2d to 9th; thence to Antwerp, and return to the United States in steamers sailing in the second week of August. The committee has entrusted all the final details to Henry Gaze & Sons, at 113 Broadway, N.Y., who have been in the tourist business since 1844, and can warrant complete satisfaction. All deposits should be made payable to this firm and they may be written to for further information and literature.

---

DELEGATES TO THE INTERNATIONAL CONGRESS OF THE MEDICAL PRESS IN PARIS.—There have been some changes in the delegation to the Medical Press Congress to be held at Paris, July 26, 27, 28, 29, 1900, since the first announcement. The following is a revised list:

From the Association of American Medical Editors: Dr. J. M. Mathews, Dr. H. Horace Grant, Louisville, Ky.; Dr. George F. Butler, Dr. George H. Simmons, Chicago; Dr. C. F. Taylor, Dr. H. A. Hare, Philadelphia; Dr. Dillon Brown, Dr. Daniel Lewis, New York; Dr. Thos. H. Hawkins, Denver, Colo.; Dr. Henry W. Coe, Portland, Ore.

The American Medical Publishers' Association: Dr. J. C. Culbertson, Cincinnati, Ohio; Mr. J. MacDonald, New York City; Dr. Ferdinand King, New York City; Mr. Charles Wood Fassett, St. Joseph, Mo.; Dr. Landon B. Edwards, Richmond, Va.

The Medical Press Club of the Mississippi Valley: Dr. I. N. Love, St. Louis, Mo.; Dr. Frank P. Norbury, Jacksonville, Ill.; Dr. Alexander J. Stone, St. Paul, Minn.; Dr. John Punton, Kansas City, Mo.; Dr. Hanau W. Loeb, Dr. C. G. Chaddock, Dr. O. F. Ball, St. Louis.

Titles of papers for the Congress should be sent to Dr. R. Blondel, 8 Rue de Castellane, Paris.

